# Case report: Dysphagia after COVID-19 infection in a stroke patient—Is neurostimulation a potential management?

**DOI:** 10.3389/fneur.2023.1126390

**Published:** 2023-03-06

**Authors:** Ting-Yu Lin, Peng-Chieh Shen, Shao-An Lee, Shu-Ming Yeh, Ke-Vin Chang, Tyng-Guey Wang

**Affiliations:** ^1^Department of Physical Medicine and Rehabilitation, Lo-Hsu Medical Foundation, Inc., Lotung Poh-Ai Hospital, Yilan City, Taiwan; ^2^Department of Physical Medicine and Rehabilitation, National Taiwan University College of Medicine, Taipei, Taiwan; ^3^Department of Physical Medicine and Rehabilitation, National Taiwan University Hospital, Taipei, Taiwan; ^4^Center for Regional Anesthesia and Pain Medicine, Wang-Fang Hospital, Taipei Medical University, Taipei, Taiwan

**Keywords:** COVID-19, dysphagia, repetitive transcranial magnetic stimulation, brain plasticity, neurostimulation

## Abstract

A 90-year-old man with stroke was weaned from tube feeding 4 months after stroke onset. However, he had a coronavirus disease 2019 (COVID-19) infection after 2 months and suffered from drastically worsened oropharyngeal dysphagia that required a reinsertion of the nasogastric tube. A videofluoroscopic swallowing study revealed poor bolus oral transit, significantly delayed swallowing reflex, reduced pharyngeal movements, and insufficient cough response. Repetitive transcranial magnetic stimulation and neuromuscular electrical stimulation were applied, in addition to conventional swallowing training. The feeding tube was removed after 20 treatment sessions. Clinicians should be aware of the risk of dysphagia after COVID-19 infection in patients with underlying neurological diseases. The management of post-COVID-19 dysphagia has not yet been fully established. Repetitive transcranial electrical stimulation combined with neuromuscular electrical stimulation may be used as an auxiliary intervention in specific cases.

## Introduction

As of September 2022, there had been over 600,000,000 confirmed cases of the coronavirus disease 2019 (COVID-19) and over 6,500,000 deaths, according to the World Health Organization (1). The severity and clinical manifestation of the disease vary widely among individuals (2, 3). Dysphagia has been reported as a sequela of COVID-19 (4, 5). Identified risk factors include pneumonia, acute respiratory distress syndrome, intubation, and old age (6). However, dysphagia can develop in non-intubated patients (7). A questionnaire screening found that 7% of non-critical, hospitalized patients with COVID-19 expressed self-perceived dysphagia after the acute phase (8). The exact mechanism of dysphagia development and persistence after COVID-19 is yet to be determined, in addition to the concerns with uncertainties in appropriate management.

Dysphagia is a common comorbidity of stroke and is associated with aspiration, pulmonary complications, malnutrition, prolonged length of hospital stay, increased healthcare expenditure, and even mortality (9–11). Conventional dysphagia training includes sensory stimulation, oral/facial/pharyngeal muscle strengthening, and swallowing maneuver education. Newer therapeutic techniques aimed at promoting neuroplasticity and recovering swallowing function have received considerable attention. Repetitive transcranial magnetic stimulation (rTMS) targets the central oropharyngeal cortex, whereas neuromuscular electrical stimulation (NMES) excites the peripheral nervous system (12). The respective and combined efficacy of these two approaches in improving poststroke dysphagia has been demonstrated (13, 14).

We present the case of a patient with stroke who experienced drastically worsened oropharyngeal dysphagia following COVID-19 infection. After conventional swallowing training, rTMS, and NMES, excellent patient outcomes were achieved.

## Case report

A 90-year-old man with hypertension, type 2 diabetes, and chronic kidney disease had cerebral infarction in the left medial temporal lobe on January 2, 2022. The patient was admitted for poststroke rehabilitation 3.5 months after onset. Prior to the stroke, the patient was able to walk independently in the community with a single cane and did not report any symptom of dysphagia. On admission (March 16, 2022), the patient had clear consciousness, but was bedridden due to right hemiplegia. The breathing pattern was smooth without supplemental oxygen. The patient was fed through a nasogastric tube with a functional oral intake scale (FOIS) score of 1. Clinical swallowing evaluation revealed reduced tongue motor skills, weak spontaneous cough, and grade I right central-type facial palsy. The gag reflex was normal bilaterally and velar elevation was symmetric. Mildly delayed swallowing reflex with a cough response was recorded during the 3-ounce water swallowing test. Two weeks after the patients was admitted (March 22, 2022), a fiberoptic endoscopic evaluation of swallowing test reported no aspiration of liquid or soft foods, but residue of soft foods in the vallecula. One week after (April 1, 2022), a videofluoroscopic swallowing study (VFSS) demonstrated a delayed swallowing reflex and premature leakage (Supplementary Video 1). The patient aspirated thin barium with a cough response. There was a slight bolus retention in the vallecula and piriform sinuses. The tests indicated that the patient had adequate swallowing ability with only minimal aspiration of thin liquid; therefore, his nasogastric tube was removed on April 1, 2022 and a modified diet was prescribed. On discharge, the patient could finish a meal by himself and consume adequate thickened liquid (FOIS: 5).

**Figure 1:**
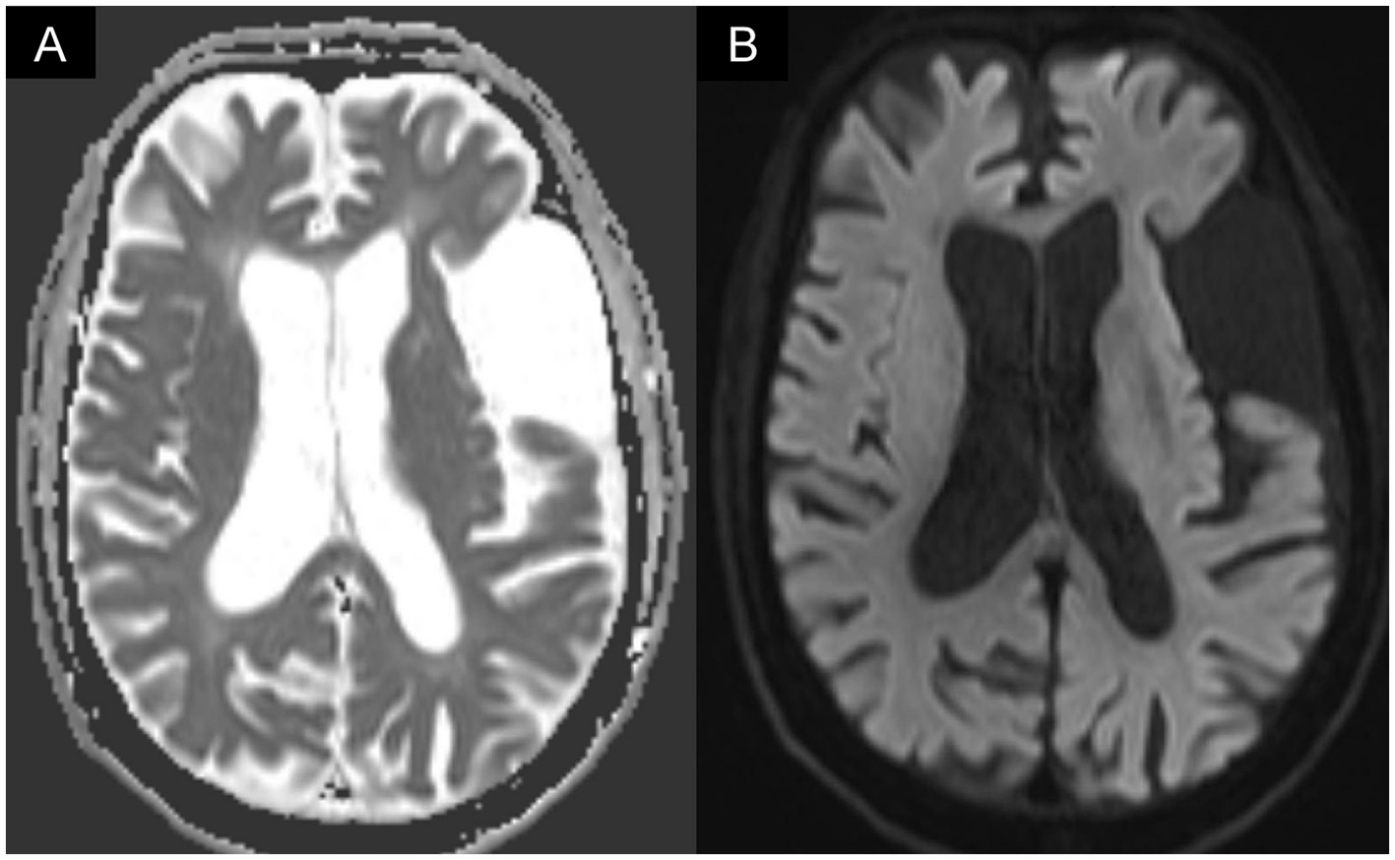
Brain MRI showed **(A)** increased signal intensity on diffusion-weighted imaging (DWI) in the left temporal lobe, which corresponded to **(B)** decreased apparent diffusion coefficient (ADC) value. The MRI image was compatible with post-stroke encephalomalacia but found no evidence of new infarction.

The patient had a fever of 38°C, cough with sputum, and rhinorrhea on June 19, 2022 (2 months after discharge), and tested positive for COVID-19. Oral antiviral molnupiravir 800 mg every 12 h was given for 5 days (June 19–June 24, 2022), and oxygen *via* a nasal cannula was provided temporarily. Fever subsided since June 22. However, owing to frequent choking and coughing, the nasogastric tube was reinserted 1 week after the patient contracted the virus. By 3 weeks after the COVID-19 infection, the patient was free of upper respiratory symptoms except for coughing when choked on his own saliva or small sips of water. The patient was admitted to our rehabilitation ward 1.5 months after the COVID-19 infection (August 2, 2022) for swallowing training. Upon admission, the patient exhibited good orientation and engaged in fluent conversations. Brunnstrom's staging and muscle strength of the patient's right limbs remained the same as they were during the previous admission; however, the patient's trunk muscles became weaker, and maintaining an upright sitting position was difficult. Excessive saliva drooling and a wet voice were observed. The patient's swallowing reflex was remarkably delayed. Gag reflexes were diminished bilaterally. Brain magnetic resonance imaging (MRI) showed no new insults (Figure 1). On August 8, 2022, a VFSS revealed severe oropharyngeal dysphagia (Supplementary Video 2). Difficulty in oral phase bolus transfer, premature oral leakage, remarkably delayed swallowing reflex, inadequate hypolarynx complex elevation, large amounts of bolus accumulation in the vallecular/pyriform sinuses, and residue spillage were shown. In addition, the patient aspirated both thin and thick barium. Cough responses although present, were weak.

Swallowing therapy with neurostimulation was afterward initiated. Training sessions began with rTMS. A magnetic stimulator (MagVenture; NeuroStar) delivered 500 pulses of 10 Hz stimulation at a 90% resting motor threshold to bilateral phayngeal cortices using a figure-of-eight coil. The rMT was identified as the intensity that produced motor-evoked potentials of 50 μV at least five out of ten times on mylohyoid electromyographic recordings. The rMT for each hemisphere was determined separately. Coil position was marked on an elastic cap that the patient wore during each session. Immediately after rTMS, the patient received a one-on-one swallowing training with a speech-language pathologist, during which sensory stimulation using NMES (Intelect; VitalStim) were employed along with swallowing muscles strengthening. The VitalStim device consists of four bipolar electrodes and were placed on both sides of the midline of the anterior neck. The top ones were situated at the level of the hyoid bone and the bottom ones at the level of the thyroid notch. The stimulation pulse was set at frequency of 80 Hz and wave amplitude of 12 mA. The 1-h-long program was administered 5 days per week for 2 weeks. The patient showed improved oral movements, with reduced drooling, better laryngeal elevation, and stronger volitional cough. The oral intake of thickened liquids and pureed foods was attempted under supervision. The chin tuck maneuver and supraglottic swallowing were used. We observed less post-swallowing choking and greater swallowing endurance. Therefore, the patient was discharged on August 26, 2022, and the management plan shifted to out-patient rehabilitation. At that time, the nasogastric tube was still necessary for nutritional requirements, which could not be met by oral intake (FOIS: 3).

After returning home, the patient continued the swallowing exercises as instructed by the rehabilitation team, which included effortful swallow, Shaker exercise, chin tuck against resistance, and expiratory muscle strengthening. The patient's swallowing ability improved daily, alongside increased oral intake with fewer choking episodes. Based on improved swallowing function, the patient was readmitted on September 22, 2022 in an attempt to wean the patient from tube feeding. Another 10 sessions of swallowing training, containing rTMS and NMES, were administered. A VFSS performed on October 7, 2022 showed good bolus transit, less premature leakage, delayed swallowing reflex, good hypopharynx elevation, penetration in thin and thick barium, trace aspiration in thin barium, and limited residue (Supplementary Video 3). With satisfactory swallowing function and adequate oral intake, the patient's nasogastric tube was removed on October 7, 2022, which was the 110th day after the patient's COVID-19 infection.

**Figure 2:**
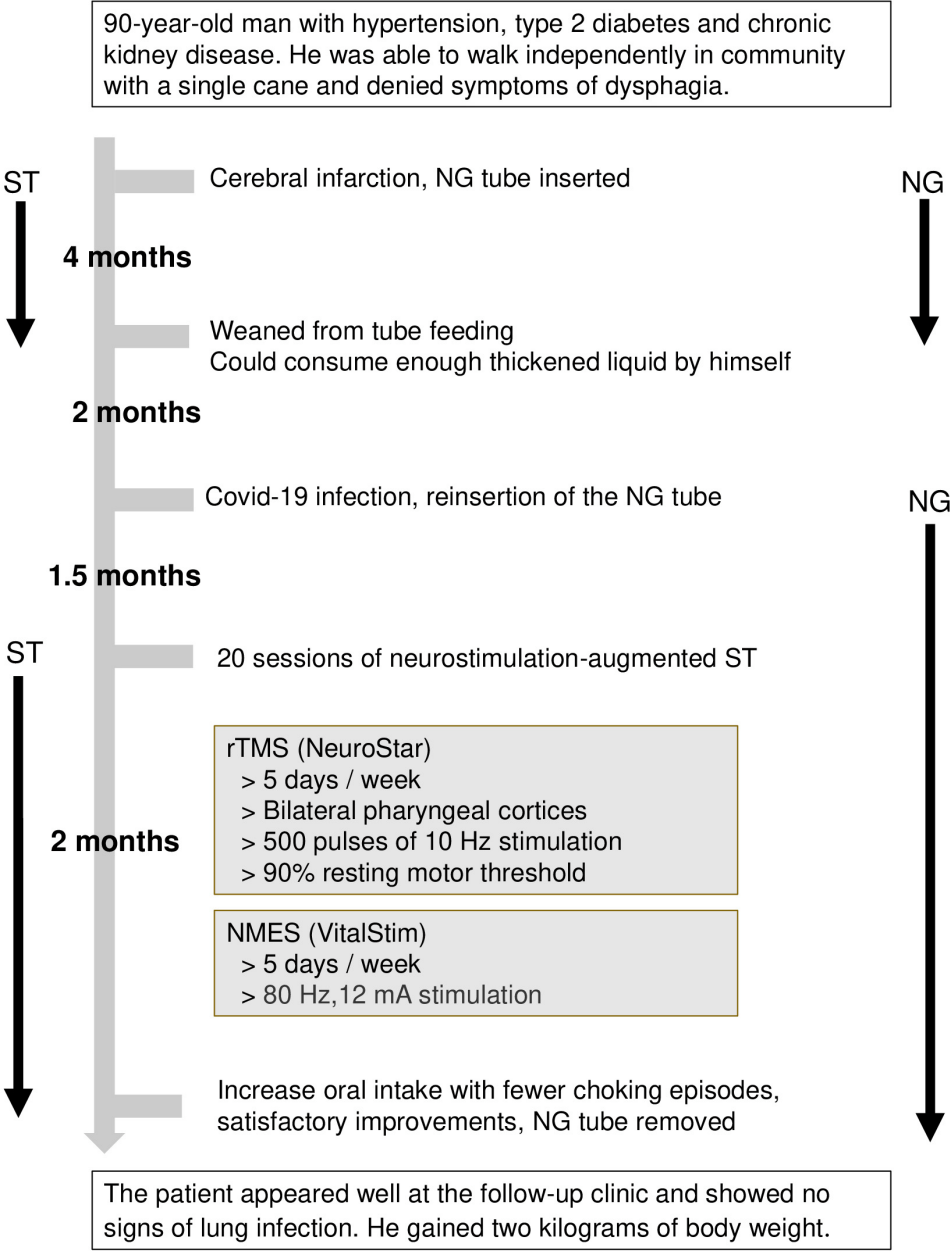
Clinical course of the patient. NMES, neuromuscular electrical stimulation; rTMS, repetitive transcranial magnetic stimulation; ST, swallowing therapy.

One month after the removal of the nasogastric tube, the patient presented in a healthy state at the follow-up clinic and showed no lung infection. In addition, the patient gained 2 kg weight after the last discharge. The clinical course is summarized in Figure 2, and the major findings of the swallowing assessments are outlined in Table 1.

**Table 1:** Findings of the VFSS studies.

**VFSS findings**	**Before COVID-19 infection (April 1st)**			**After COVID-19 infection (August 8th)**			**After swallowing therapy (October 7th)**		
	**Thin**	**Thick**	**Paste**	**Thin**	**Thick**	**Paste**	**Thin**	**Thick**	**Paste**
**Oral phase**									
Oral stasis	(–)	(–)	(–)	(+)	(+)	(+)	(+)	(–)	(–)
Premature oral leakage	(+)	(+)	(+)	(+)	(+)	(–)	(+)	(+)	(+)
**Pharyngeal phase**									
Hypohyoid elevation	Adequate	Adequate	Adequate	NA	Poor	NA	Adequate	Adequate	Adequate
Vallecular stasis	(+), < 1/2	(–)	(–)	NA	(+), >1/2	NA	(+), >1/2	(+), < 1/2	(+), < 1/2
Pyriform stasis	(–)	(–)	(–)	NA	(+), >1/2	NA	(–)	(–)	(–)
Premature spillage	(+)	(–)	(–)	NA	(+)	NA	(+)	(–)	(–)
PAS	7	1	1	NA	7	NA	8	2	1
Swallow reflex (seconds)	3	8	5	(–)	>10	(–)	>10	4	8

Hypothyroid elevation was defined as adequate when there was complete airway closure during bolus swallow whereas poor hypohyoid elevation indicated incomplete airway closure and protection. Vallecular/pyriform stasis was expressed as more than half (>1/2) or less than half (< 1/2) of the space of the vallecular/pyriform sinuses.

PAS, penetration-aspiration scale; NA, not available.

## Discussion and conclusion

In this case report, we presented the development of post-COVID-19 dysphagia in a patient with stroke and the patient's subsequent recovery using several swallowing assessments. The patient was weaned off tube feeding 4 months after the stroke. However, he had COVID-19 infection after 2 months and reported worsened oropharyngeal dysphagia. A nasogastric tube was inserted to maintain safe enteral nutrition. Twenty sessions of swallowing therapy augmented with neurostimulation were administered. The feeding tube was removed 3.5 months after COVID-19 infection.

Newly-diagnosed oropharyngeal dysphagia was found in 35.3% of hospitalized patients with COVID-19 (15). Dziewas et al. (16) postulated several etiologies of COVID-19-associated dysphagia, including encompassing stroke, encephalitis, critical illness neuropathy, Guillain-Barré syndrome (GBS), and skeletal muscle injury. In our case, recurrent stroke was excluded by brain MRI. The patient did not present with limbs twitching, speech disturbance, vomiting, altered consciousness, unusual behaviors or personality changes. Although cerebrospinal fluid analysis and electroencephalography were not performed, encephalitis and non-convulsive seizures were unlikely. Muscle strength and sensation across the four limbs remained constant throughout the disease course, which is incompatible with critical illness neuropathy, GBS or its pharyngeal-cervical-brachial variant which can initially present with swallowing difficulties. Myositis was unlikely because the patient did not have muscle pain and had normal serum creatinine kinase levels.

Cranial nerve dysfunction is a possible explanation for the marked deterioration in swallowing function after COVID-19 infection in our case. Dysosmia and dysgeusia are common complications of COVID-19. The virus enters the human body by binding with angiotensin-converting enzyme II cell receptors, which are expressed in the tongue, oral mucosa, and olfactory epithelium (17). Neurotropism, the direct viral invasion of nerves, is the most reported pathophysiology of cranial nerve involvement in COVID-19 (18). Other speculated causes include involvement of the central nervous system, focal immune response, and inflammatory reaction (18, 19). Gag reflex disappeared and laryngeal sensation reduced after the COVID-19 infection in our patient, which might be a presentation of glossopharyngeal and vagal neuropathies. Damage to the trigeminal and hypoglossal nerves impairs bolus formation and propulsion. Furthermore, breathing-swallowing coordination is fundamental for protecting the lower airway. Symptoms, such as coughing, sneezing, and shortness of breath, can hamper this predetermined rhythm. In addition, malnutrition and deconditioning from an acute infection may play a role in the patient's dysphagia, as the patient had a concurrent decline in physical function. Fatigue can persist after the acute phase of a COVID-19 infection (20). In this patient, eternal nutrition and body weight were promptly supported by tube feeding. Progress in exercise endurance and transfer skills was made during admission; however, the progress was to a lesser extent compared to swallowing ability.

Dysphagia among non-intubated patients with COVID-19 is generally self-limiting (7). An observational cohort study by Archer et al. (21) found that in 70.7% of patients referred for post-COVID-19-associated swallowing problems, the swallowing problems fully resolved on discharge. However, they also pointed out that patients with preexisting neurologic diagnoses were prone to experience persistent dysphagia in the absence of mechanical ventilation. Lee et al. (22) described that a new-onset swallowing difficulty in a patient with Parkinson's disease was so severe that the patient continued to rely on tube feeding 2 months after the viral infection. In the present case, the patient sustained an infarction and developed poststroke dysphagia. Nonetheless, through rehabilitative endeavors, the feeding tube was successfully discontinued 4 months after stroke onset. The dramatic reduction of swallowing function following COVID-19 infection could be attributed to the failure of compensatory techniques when prior deficits are coupled with COVID-19-related neuromuscular dysfunctions, respiratory distress, and fatigue.

In recent years, neurostimulation has been extensively studied for its ability to modulate neuroplasticity and maximize functional recovery. Changes to the neural network could be induced by applying stimulation directly to the motor cortex (rTMS) or through peripheral somatosensory stimulations (NMES) (23, 24). Promising outcomes were shown in the management of numerous neurological/psychological diseases with rTMS (25). Furthermore, there is growing evidence that it is a validated approach to treat poststroke dysphagia (13). The effectiveness of rTMS is significantly greater when administered alongside conventional swallowing therapy than when being administered as a stand-alone treatment (26). However, stimulation protocols and their respective results are diverse. In particular, a randomized controlled study performed by Park et al. (27) illustrated bilateral stimulation produced better and faster improvements than unilateral stimulation. NMES is effective in treating dysphagia in patients with and without stroke (28, 29). A report found a superior recovery of poststroke dysphagia in the rTMS plus NMES group than in the NMES group (14). No study has mentioned using neurostimulation for the treatment of dysphagia after COVID-19 infection. As both the central and peripheral nervous systems could be involved in prolonged dysphagia in our case, we adopted rTMS and NMES to optimize the patient's recovery. Surprisingly, the results were good. The extent to which neurostimulation contributed to the favorable outcome in our patient was uncertain. The intensity of the neurostimulation protocol (500 pulses of rTMS combined with NMES for 20 days in our case) may be crucial to its effectiveness. Nonetheless, this report may provide some clues to the etiology and treatment of post-COVID-19 dysphagia.

In summary, we emphasized the risk of dysphagia after COVID-19 infection in patients with premorbid neurological conditions and swallowing difficulties. The multifactorial detrimental effects of COVID-19 can have serious consequences in vulnerable populations. Intensive rehabilitation yielded favorable outcomes in this case. The therapeutic potential of neurostimulation in post-COVID-19 dysphagia is worth further investigation.

## Data availability statement

The original contributions presented in the study are included in the article/Supplementary material, further inquiries can be directed to the corresponding author.

## Ethics statement

Ethical review and approval was not required for the study on human participants in accordance with the local legislation and institutional requirements. The patients/participants provided their written informed consent to participate in this study. Written informed consent was obtained from the individual(s) for the publication of any potentially identifiable images or data included in this article.

## Author contributions

S-MY and T-GW contributed to conception and design of the study. T-YL wrote the first draft of the manuscript. S-MY, K-VC, and T-GW reviewed and edited the manuscript. P-CS and S-AL prepared the table, figure, and videos. All authors read and approved the submitted version.
